# Percutaneous isolated hepatic perfusion (chemosaturation) with melphalan following right hemihepatectomy in patients with cholangiocarcinoma and metastatic uveal melanoma: peri- and post-interventional adverse events and therapy response compared to a matched group without prior liver surgery

**DOI:** 10.1007/s10585-020-10057-9

**Published:** 2020-10-09

**Authors:** C. L. A. Dewald, L. S. Becker, S. K. Maschke, T. C. Meine, T. A. Alten, M. M. Kirstein, A. Vogel, F. K. Wacker, B. C. Meyer, J. B. Hinrichs

**Affiliations:** 1 Department of Diagnostic and Interventional Radiology, Institute for Diagnostic and Interventional Radiology, Hannover Medical School, Carl-Neuberg-Str. 1, 30625 Hannover, Germany; 2 Department of Gastroenterology, Hepatology and Endocrinology, Hannover Medical School, Hannover, Germany

**Keywords:** Chemosaturation, Hemihepatectomy, Melphalan, Metastatic uveal melanoma, Cholangiocarcinoma, Percutaneous locoregional therapy

## Abstract

To evaluate feasibility, frequency and severity of peri-procedural complications and post-procedural adverse events (AEs) in patients with advanced cholangiocarcinoma or liver metastasis of uveal melanoma and prior hemihepatectomy undergoing chemosaturation percutaneous hepatic perfusion (CS-PHP) and to analyze therapy response and overall survival compared to a matched group without prior surgery. CS-PHP performed between 10/2014 and 02/2018 were retrospectively assessed. To determine peri-procedural safety and post-procedural adverse events, hospital records and hematological, hepatic and biliary function were categorized using Common Terminology Criteria for Adverse Events (CTCAE) v5.0 (1–5; mild-death). Significance was tested using Wilcoxon signed-rank and Mann–Whitney U test. Kaplan–Meier estimation and log-rank test assessed survival. Overall 21 CS-PHP in seven patients (4/7 males; 52 ± 10 years) with hemihepatectomy (group^hemihep^) and 22 CS-PHP in seven patients (3/7 males; 63 ± 12 years) without prior surgery (group^noresection^) were included. No complications occurred during the CS-PHP procedures. Transient changes (CTCAE grade 1–2) of liver enzymes and blood cells followed all procedures. In comparison, group^hemihep^ presented slightly more AEs grade 3–4 (e.g. thrombocytopenia in 57% (12/21) vs. 41% (9/22; p = 0.37)) 5–7 days after CS-PHP. These AEs were self-limiting or responsive to treatment (insignificant difference of pre-interventional to 21–45 days post-interventional values (p > 0.05)). One patient in group^hemihep^ with high tumor burden died eight days following CS-PHP. No deaths occurred in group^noresection^. In comparison, overall survival after first diagnosis was insignificantly shorter in group^noresection^ (44.7(32–56.1) months) than in group^hemihep^ (48.3(34.6–72.8) months; p = 0.48). The severity of adverse events following CS-PHP in patients after hemihepatectomy was comparable to a matched group without prior liver surgery. Thus, the performance of CS-PHP is not substantially compromised by a prior hemihepatectomy.

## Introduction

Surgical resection is an important treatment option for intrahepatic cholangiocarcinoma (iCCA) and metastatic uveal melanoma (UM) [1]. Determined by extent of tumor and anatomical location, liver resections vary from atypical resection to hemihepatectomy. Despite the curative intent of surgical resection, tumor recurrence in iCCA is a common problem with reported rates of up to 50% [2, 3] and in metastatic UM, recurrence rates of up to 80% are described [4, 5]. Tumor relapse is often challenging, as re-resection might not be suitable due to small fraction of left functional liver tissue and due to more challenging anatomical situs. According to current guidelines for iCCA, locoregional therapies can be considered after first-line chemotherapy and chemosaturation percutaneous hepatic perfusion (CS-PHP) already showed encouraging results in early studies [1, 6]. Concerning inoperable metastasized UM, no standard of care is available and current guidelines recommend ablation, infusion, perfusion and/or embolization therapies tailored to number and location of the metastases [7]. As in metastatic UM the liver is often the first and only site of metastases [8], liver directed therapies such as CS-PHP are of increasing relevance. Nonetheless, dedicated research investigating CS-PHP following liver resection is missing.

CS-PHP with melphalan is an innovative locoregional therapy for hepatic malignancies. The aim of CS-PHP is to control tumor growth, palliate symptoms and extent survival. Several studies have demonstrated efficacy in iCCA and UM and confirmed safety of the procedure [1, 9–11]. CS-PHP is taking advantage of the unique hepatic anatomy: high dose melphalan is administered via a catheter placed in the hepatic artery and thus, provides a non-diluted chemoperfusion of the diseased liver parenchyma. To prevent systemic damage caused by the melphalan-enriched blood, a double balloon catheter is placed in the inferior vena cava (IVC), which occludes the IVC above and below the confluens of the liver veins. The venous hepatic blood is extracted through the double balloon catheter, filtered via an extracorporeal melphalan specific filtration system and returned to the circulation through a sheath in the right jugular vein [11, 12].

In patients following resection of hepatic tissue, CS-PHP might be more complex and potentially hazardous. First, the anatomical changes following liver resection due to scarring, compensatory hypertrophy and changes of the venous drainage to the vena cava might compromise a safe positioning of the catheters. Second, the toxic effect of melphalan on the overall reduced liver parenchyma after hepatic resection might be increased. Thus, the purpose of this study was to evaluate frequency and severity of peri-procedural complications and post-procedural adverse events in patients with right hemihepatectomy undergoing CS-PHP and to compare these to a matched group without prior surgery.

## Material and methods

### Patient selection

The local ethics committee approved this retrospective study. In our tertiary care referral center, an interdisciplinary liver tumor board reaches treatment decisions for all patients with primary or secondary intrahepatic malignancies. From 10/2014 to 02/2018, 52 patients were scheduled for CS-PHP as last line treatment option and underwent 112 procedures (patient characteristics see Table 1). Among these 52 patients were seven patients with prior hemihepatectomy, who underwent 21 CS-PHP (group^hemihep^), and were included in this study. No patient with prior hemihepatectomy undergoing CS-PHP was excluded.

**Table 1:** Patient demographics of the original study group

Age at first CS-PHP (years)^a^	60 (53–79)
Gender	36.5% m 63.5% f
Primary malignancy	24 UM 14 CCC 6 HCC 2 CRC 1 EC 1 BRCA 2 NEC 2 PAAD
Pre-interventional tumor load (%)^a^	9.78 (3.11–27.12)

*UM* uveal melanoma *CCC* cholangiocarcinoma *HCC* hepatocellular carcinoma *CRC* colorectal carcinoma, *EC* endometrial carcinoma, *BRCA* breast invasive carcinoma, *NEC* neuroendocrine carcinoma, *PAAD* pancreas adenocarcinoma

^a^Values are presented in median and interquartile range

Additionally, we defined seven patients (undergoing 22 CS-PHP) without hepatic resection as control group (group^noresection^). In order to achieve comparable patient collectives, group^hemihep^ and group^noresection^ were matched by primary tumor, age, sex, number of consecutive CS-PHP and pre-interventional level of lactate dehydrogenase (LDH). Requirements for CS-PHP were sufficient hematological (haemoglobin > 8 g/dL; white blood count > 2 thsd/μL; platelets > 50 thsd/μL), hepatic (bilirubin ≤ 3 × upper limit of normal, maximum Child–Pugh A) and renal function (serum creatinine > 60 µmol/L). Contraindications included a recent history of transient ischemic attacks, heart failure with a left ventricular ejection fraction < 40% or significant chronic obstructive or restrictive pulmonary disorder. LDH, indicating tumor burden, should not exceed 500 IU/L. ECOG performance status must score 0 or 1 before CS-PHP. All patients gave written consent before CS-PHP.

Part of this study population has previously been reported [11, 13]. These articles dealt with safety and efficacy of the second-generation CS-PHP, whereas this study focuses on peri- and post-interventional adverse events and therapy response of patients with previous right hemihepatectomy compared to a matched group without prior liver surgery.

### Data acquisition

Complications were observed during defined periods. In the peri-procedural period (start of general anesthesia to transfer to intensive care unit, adverse events (AEs) are more likely to be linked directly to the CS-PHP procedure. During the post-procedural period (1–2 days on intensive care unit), 5–7 days following CS-PHP (in-patient stay on regular ward) and during the subsequent 21–45 days (first control as outpatient), AEs are more likely to be related to systemic exposure of chemotherapeutic melphalan. Clinical reports regarding the hospital stay and follow up examinations were screened. Haematological parameters including full blood count and international normalized ratio (INR) as well as alanine transferase, aspartate transferase, gamma-glutamyl-transferase, alkaline phosphatase, total bilirubin and albumin indicating the liver function, LDH, creatinine as kidney function parameter, c-reactive protein and MELD-Scores were analysed. Values were assessed and classified using Common Terminology Criteria for Adverse Events (CTCAE) Version 5.0. CTCAE is a descriptive terminology used for AE reporting. A severity scale is provided for each AE term (grades 1–5; mild-death). The assessment was performed by one radiologist (C.L.A.D.), blinded to the patient allocation to group^hemihep^/group^noresection^. Pre-interventional measurements were considered baseline.

Either CT or MRI was performed before and within three months after CS-PHP. Treatment response was measured according to the RECIST 1.1 [14]. Overall survival (OS) was determined from initial diagnosis and first CS-PHP until last follow-up or death, whichever occurred first.

### CS-PHP-Procedure

**Fig. 1:**
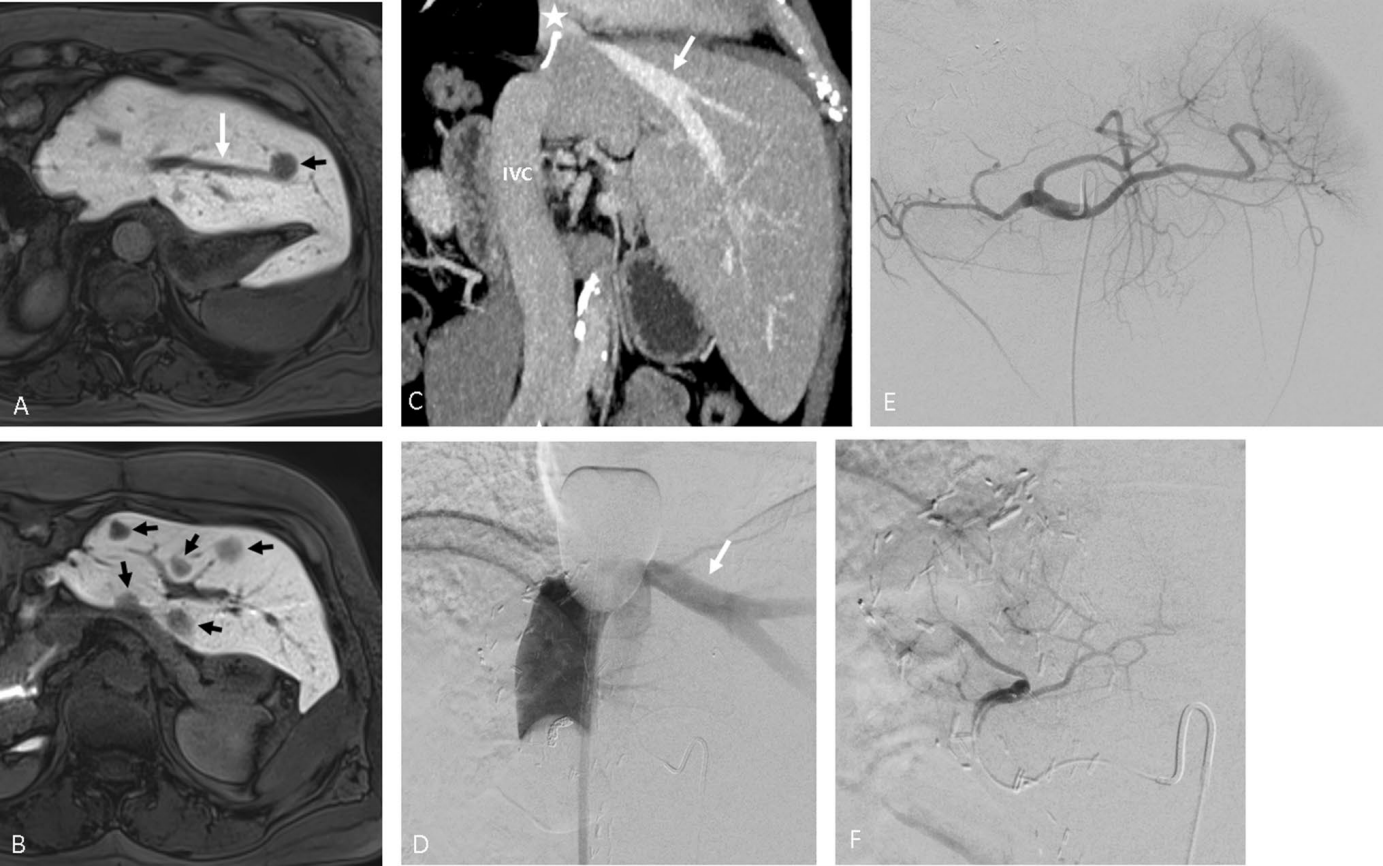
Gd-EOB DTPA-enhanced MRI of a patient with prior right hemihepatectomy. The multifocal hepatic metastasis of uveal melanoma in the left liver (black arrows) are clearly depicted in the delayed transversal T1 weighted phase (**a**, **b**). A coronal CT scan (**c**) gives an overview of the postoperative situs following right hemihepatectomy. The star (*) marks the confluens of the liver veins (white arrow) close to the resection margin. **d** Displays the retrograde injection of contrast agent during CS-PHP to verify correct placement of the double balloon catheter. The cranial balloon of the double balloon catheter is placed in the cavoatrial junction in close proximity to the resection margin. The caudal balloon is placed below the confluens of the liver veins. No leakage is visible while the left liver vein is opacified (white arrow). **e** Presents an overview angiography of the celiac trunk with a small left hepatic artery. The catheter used for administration of melphalan is advanced to the left hepatic artery (**f**). IVC = inferior vena cava

All procedures were performed in an angio suite under general anaesthesia due to the lengths of the intervention (mean 164 ± 52 min) and due to the haemodynamic changes, which are common with the transient inferior vena cava (IVC) occlusion and blood filtration [15]. In CS-PHP, an appropriate sheath is inserted through the femoral artery and a catheter is placed in the hepatic artery to provide a chemoperfusion with high dose melphalan of the supplied liver parenchyma. In order to prevent systemic exposure of the toxic melphalan, a double balloon catheter, inserted through the femoral vein, is placed in the IVC. The cranial balloon is inflated close to the cavoatrial junction and the caudal balloon is inflated in the subhepatic segment of the IVC, below the confluens of the hepatic veins (Fig. 1). The double balloon catheter is equipped with multiple side holes. An extracorporeal pump extracts the melphalan-enriched blood into a dedicated filtration system (Delcath system’s second-generation hemofiltration system), which separates melphalan from the venous blood with a filtration rate of up to 93–96% [15–17]. The extracorporeal circuit is completed by the return of the blood via a sheath in the jugular vein. In order to maintain an activated clotting time above 500 s, which is essential for safe extracorporeal hemofiltration, heparin is administered as needed. The melphalan dosage used in boths group^hemihep^ and group^noresection^ was bodyweight dependent, 2,5–3 mg/kg ideal body weight up to a maximum dose of 220 mg of melphalan, dissolved in a 500 cc solution. The chemotherapeutic agent is infused in aliquots of each 100 cc at a rate of 0.4 ml/s, in between which an angiogram is performed to ascertain proper flow in the hepatic artery of interest.

24–72 h following CS-PHP, patients received single-shot antibiotics and granulocyte-colony stimulating factor.

### Statistical analysis

Descriptive statistical analysis of the study data was performed. Survival was assessed using Kaplan–Meier estimation and log-rank test. Related data were tested for significance using non-parametric pairwise Wilcoxon signed-rank test and Mann–Whitney U test was performed for group comparison. Contingency table analysis was performed using chi-square test. Level of significance was set to p < 0.05. Statistical analyses were performed using commercially available software (JMP 15, SAS Institute). Values are presented in mean (and standard deviation) or median (and interquartile range).

## Results

Overall, 21 CS-PHP in seven patients (52 ± 10 years, four men and three women) with prior right hemihepatectomy as well as 22 CS-PHP in seven patients (63 ± 12 years, three men and four women) without prior hepatic resection were included in this study. Prevalence of the underlying tumor entities were as follows: in group^hemihep^, five patients with consecutive 14 CS-PHP suffered from iCCA and two patients underwent six CS-PHP in consequence of a metastasized UM. In group^noresection^, five patients with iCCA and 17 conducted CS-PHP as well as two patients with UM and five successful interventions were included. The patients median LDH values prior to the first CS-PHP were 320 (232–378) U/L in group^hemihep^ and 242 (236–333) U/L in group^noresection^ (p = 0.95). Detailed patients` demographics, clinical and interventional parameters are displayed in Table 2.

**Table 2:** Patient demographics, clinical and interventional parameters of group^hemihep^ and group^noresection^

Patients	Primary malignancy	Age at first CS-PHP (years)	Sex	Number of CS-PHP	LDH (U/I) previous to first CS-PHP	Hemi- hepatectomy until first CS-PHP (months)	Initial diagnosis until first CS-PHP (months)
group^hemihep^							
1	iCCC	60	m	5	206	27	27
2	iCCC	59	m	1	376	13	13
3	iCCC	39	m	1	320	16	16
4	iCCC	61	f	1	233	77	78
5	iCCC	36	f	7	380	4	5
6	UM	49	f	4	230	8	83
7	UM	55	m	2	1559	90	262
group^noresection^							
1	iCCC	75	m	5	283	n.a	35
2	iCCC	81	m	2	382	n.a	10
3	iCCC	62	m	2	224	n.a	42
4	iCCC	61	f	4	233	n.a	12
5	iCCC	53	f	4	238	n.a	8
6	UM	46	f	4	242	n.a	44
7	UM	57	f	1	1064	n.a	42
group^hemihep^	71% iCCC 29% UM	52 ± 10 **	43% f 57% m	21 interventions in total	320 (232–378) *	10.5 (16–52)*	27 (14.5–80.5)*
group^noresection^	71% iCCC 29% UM	63 ± 12 **	57% f 43% m	22 interventions in total	242 (236–333) *	n.a	35 (11–42)*
p-value		0.14		0.74	0.95		0.5

iCCC  intrahepatic cholangiocarcinoma, *f*  female, *LDH*   lactate dehydrogenase, *M*   male, *UM*   uveal melanoma

*Values are presented in median and interquartile range, **Values are presented in mean and standard deviation

### CS-PHP and peri-interventional complications

There were no AEs of grade 3–5 recorded during the interventions. Hypotension and tachycardia were recorded during all procedures following inflation of the double balloon catheter and initiation of the veno-venous bypass including the filtration system and successfully managed by the anaesthesiologists. Overall, the cardiovascular fluctuations were self-limiting at the end of the procedures. Before administration of melphalan, the double balloon catheter had to be repositioned due to leakage of the cranial balloon in 3/21 interventions of three patients in group^hemihep^ and in 3/21 consecutive interventions of one patient in group^noresection^. The mean melphalan dose was 174 ± 24 mg in group^hemihep^ and 174 ± 30 mg in group^noresection^ (p = 0.47). The mean procedure time was 169 ± 27 min in group^hemihep^ and 176 ± 52 min in group^noresection^ (p = 0.69).

### Toxicity and complications

In total, 114 AEs (grade 1–5) occurred in group^hemihep^ (5.5 AEs per intervention) and 106 AEs in group^noresection^ (4.8 AEs per intervention; p = 0.68). The percentage distribution (Table 3) of the AEs grade 1–4 was comparable between group^hemihep^ and group^noresection^ with the majority of AEs being rated mild (grade one; group^hemihep^ 32% (32/114) and group^noresection^ 35% (37/106)) or moderate (grade two; group^hemihep^ 38% (44/114) and group^noresection^ 38% (40/106)).

**Table 3:** Distribution of adverse events (AEs) grade 1–5

	Group^hemihep^ n = 21 interventions		Group^noresection^ n = 22 interventions	
AEs grades 1–5 in total	114	100%	106	100%
AEs grade 1	37	32%	37	35%
AEs grade 2	44	38%	40	38%
AEs grade 3	29	25%	27	25%
AEs grade 4	3	3%	2	2%
AEs grade 5	1	1%	0	0%

**Fig. 2:**
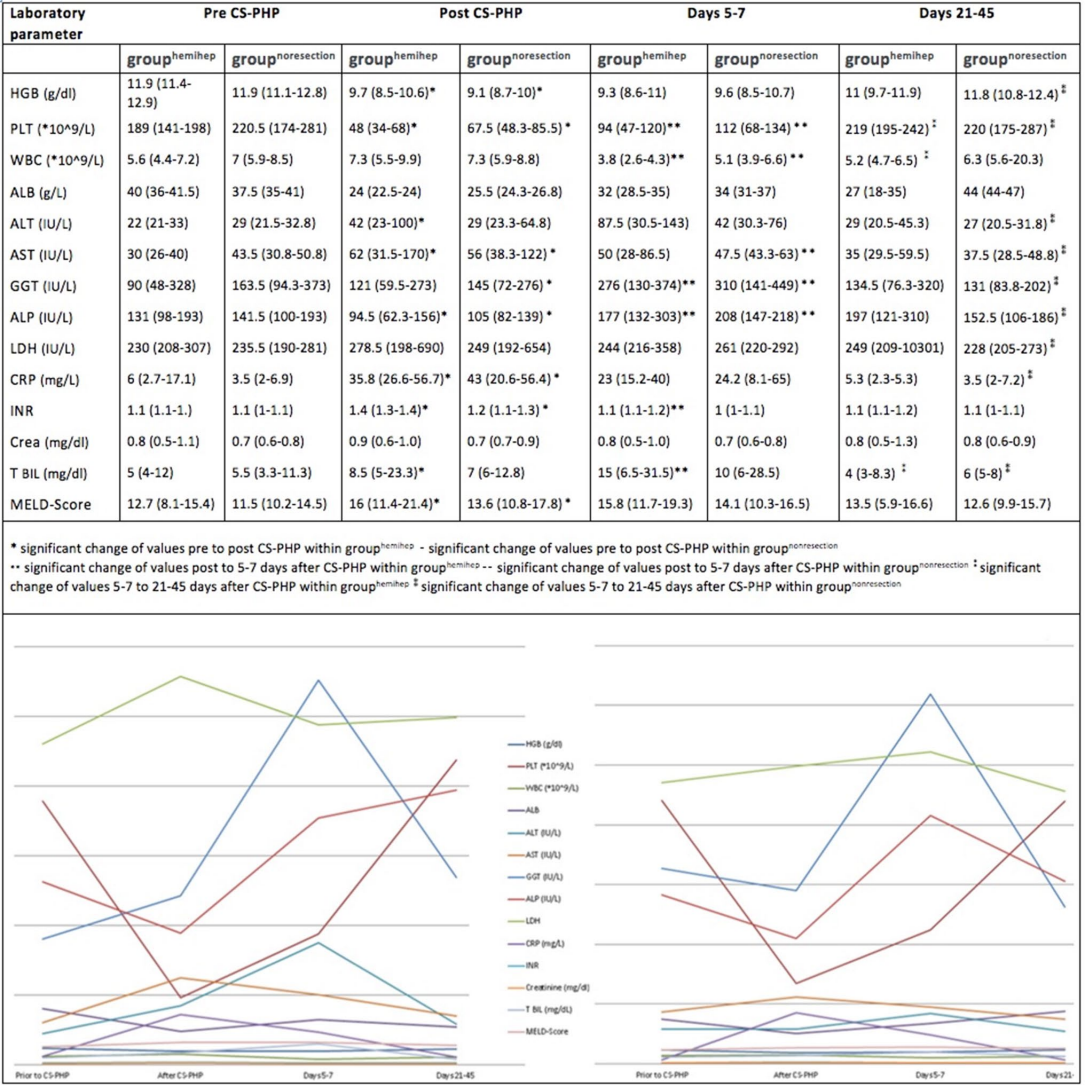
Hematological and hepatic parameters of grouphemihep and groupnoresection pre, post, 5–7 days and 21–45 days after CS-PHP in a tabular overview (median and interquartile range); simplified course of laboratory parameters below. Statistical significance of parameter changes within grouphemihep and groupnoresection has been tested (p ≥ .05). *HGB*   hemoglobin, *PLT* = platelet, *WBC*  white blood count, *ALB*  albumin, *ALT*  alanine transferase, *AST*  aspartate transferase, *GGT*  gamma-glutamyl transferase, *ALP*   alkaline phosphatase, *LDH*  lactate dehydrogenase, *CRP*  c-reactive protein, *INR*  international normalized ratio, *Crea* creatinine, *T* *BIL* = total bilirubin, *MELD-Score*  model of end stage liver disease-score

All interventions were followed by thrombocytopenia, anemia and an increase of liver enzymes. Most clinically relevant AEs accounted for toxicity-related hematologic and liver parameter changes (refer to Table 4): post-interventional thrombocytopenia grade three was common (52% (11/21) in group^hemihep^ and 36% (8/22) in group^noresection^; p = 0.36). Grade four thrombocytopenia with need for thrombocyte concentrates was observed after one procedure (5%) in each group. A reduced white blood count (WBC) equivalent to AE grade three was noted after 5–7 days in 24% (5/21) of the CS-PHP in group^hemihep^ and in 9% (2/22) of the CS-PHP in group^noresection^ (p = 0.24). Increase of liver enzymes (AE grade three) was frequent in both groups (29% (6/21) in group^hemihep^ and 18% (4/22) in group^noresection^; p = 0.73). Whereas aspartate transferase values were elevated post-interventionally, alkaline phosphatase values showed an early decrease and an increase on day 5–7. The above-mentioned changes in blood count and liver enzymes were transient in both groups–no statistical significance was found when comparing baseline values to late post-interventional (21–45 days after CS-PHP) measurements. Detailed courses of laboratory parameters are displayed in Fig. 2.

**Table 4:** Detailed listing of all common terminology criteria for adverse events grades 3–5 in both groups. Absolute and relative values (% of interventions) are presented

Type of adverse event	Group^hemihep^ n = 21 interventions					Group^noresection^ n = 22 interventions				
	Grade 3	%	Grade 4	%	% in total	Grade 3	%	Grade 4	%	% in total
Hematological/toxic										
Reduced white blood count	5	**24%**	/		24%	2	**9%**	/		9%
Thrombocytopenia	11	**52%**	1	**5%**	57%	8	**36%**	1	**5%**	41%
Anemia	3	**14%**	/		14%	3	**14%**	/		14%
Non-hematological/toxic										
Increased liver enzymes	6	**29%**	/		29%	4	**18%**	/		18%
Tumor lysis syndrome	1	**5%**	/		5%	/		/		/
Decreased coagulation factors	/		/		/	1	**5%**	/		5%
Inflammatory/infectious										
Sepsis	/		/		/	1	**5%**	/		5%
Cholangitis	/		1	**5%**	5%	2	**10%**	/		10%
SIRS	1	**5%**	/		5%	4	**18%**	/		18%
Exacerbation of urinary tract infection	/		/		/	1	**5%**	/		5%
Other										
Pleural effusion	/		/		/	1	**5%**	/		5%
Anaphylactic reaction	/		/		/	/		1	**5%**	5%
Aspiration pneumonia	/		/		/	1	**5%**	/		5%
Hypo/-hypertension	2	**10%**	1	**5%**	15%	/		/		/
Stroke; persistent symptoms	/		/		/	1	**5%**	/		5%
Stroke; transient symptoms	1	**5%**	/		5%	/		/		/
Death/Grade 5	1	**5%**			5%	/		/		/
CTCAE grade 3–4 in total					33					31

*CTCAE*  common terminology criteria for adverse events, *SIRS*  systemic inflammatory response syndrome

Non-toxic AEs grade three compromised inflammatory or infectious complications on day 5–7 after 5% (1/21) of the CS-PHP in group^hemihep^ and after 38% (8/22) of the interventions in group^noresection^ (for details refer to Table 3). Non-toxic AEs grade 4 were: cholangitis (5%;1/21) after 5–7 days and post-interventional hypotension (5%;1/21) in group^hemihep^ and a post-interventional anaphylactic reaction to protamine (5%;1/22) in group^noresection^.

A case of death (AE grade five) occurred in group^hemihep^ in a patient with hepatic metastases from uveal melanoma and a high pre-interventional tumor burden (LDH 1559 U/I) (Table 1; patient seven). The patient developed multi-organ failure as well as pancytopenia and deceased eight days after his second CS-PHP despite intensive care treatment. There was no AE grade five in group^noresection^.

### Therapy response and overall survival

In group^hemihep^, the median time from initial diagnosis to first CS-PHP was 27 (14.5–80.5) months compared to 35 (11–42) in group^noresection^ (p = 0.4). The median time from right hemihepatectomy to first CS-PHP was 10.5 (6–52) months. Following CS-PHP, the first follow up exam was performed 47 (42–60) days following CS-PHP in group^hemihep^ and 49 (43–71) days in group^noresection^ (p = 0.78). The first therapy response according to RECIST 1.1 was 100% stable disease (SD) in group^noresection^ (7/7). In the first response assessment of group^hemihep^, 43% of patients presented with SD (3/7), 14% with complete remission (1/7), 14% with partial response (1/7) and 29% (2/7) with progressive disease; one of these two patients had extrahepatic progress. Taking into account not only the first but all therapy responses, the best response was 47% SD in group^hemihep^ (4/7), 71% SD in group^noresection^ (5/7), 14% complete remission in both groups (1/7), 14% partial response in both groups (1/7) and 14% progressive disease (extrahepatic progression) in group^hemihep^ (1/7).

The median overall survival (OS) after first CS-PHP in group^noresection^ was longer (19.7 (7.5–23.8) months) than in group^hemihep^ with 9.3 (4.2–17) months (p = 0.53). When comparing mean OS (months) from initial diagnosis, group^noresection^ (44.7 (32–56.1)) had a shorter OS than group^hemihep^ (48.3 (34.6–72.8)) (p = 0.48).

## Discussion

Chemosaturation percutaneous hepatic perfusion delivers high doses of chemotherapy directly to the liver while limiting systemic toxicity via hemofiltration of the hepatic venous blood [11]. In this retrospective study, we compared the peri- and post-interventional adverse events and therapy response of CS-PHP in seven patients with prior hemihepatectomy undergoing 21 procedures (group^hemihep^) to a matched cohort of seven patients without hepatic surgery and 22 conducted interventions (group^noresection^). Our data show, that CS-PHP after hemihepatectomy has manageable toxicity comparable to a group of non-resected patients. Therefore, CS-PHP might serve as a potential last-line palliative treatment option for selected patients with cholangiocarcinoma and liverdominant metastatic uveal melanoma even after prior liver surgery.

The potential merit of locoregional liver therapies in metastatic uveal melanoma is controversially discussed and is of high importance as favorable systemic therapies are still lacking [18]. Khoja et. al analysed 29 phase II studies based on the original survival and response data and concluded, that locoregional therapy approaches present a clear numeric advantage compared to systemic therapies [19]. The use of CS-PHP in patients with liverdominant UM is based on the results of a landmark phase III randomized controlled study [9], which showed an improved progression free survival after CS-PHP versus best alternative care (BAC). Unfortunately, a high crossover of BAC patients to the CS-PHP group confounded any possible survival advantage in this study.

In the setting of unresectable iCCA, guidelines suggest locoregional therapy approaches in patients with tumor progression under first-line systemic chemotherapy [6]. CS-PHP in iCCA has so far only been evaluated in small cohorts, but presented long-lasting tumor stabilization in selected patients [1, 13, 20]. In order to provide optimal patient tailored treatment options, possible benefits over other locoregional therapy approaches (e.g. transarterial radioembolization (TARE)) need to be further evaluated.

The overall OS of our study collective is in line with the original phase III study of Hughes et al. and other current studies reporting OS ranging from 10 to 27 months [9, 20–23]. There was a trend for a shorter median overall survival in group^hemihep^, which was 9.3 months after first CS-PHP in group^hemihep^ compared to 19.7 months in group^noresection^ (p = 0.53). Of note, patients in group^hemihep^ were younger than in group^noresection^ and the first CS-PHP was performed earlier after first diagnosis than in group^noresection^. The rapid disease progression of group^hemihep^ might reflect a more aggressive tumor biology and thus might explain the trend for a shorter OS.

Comparable numbers of AEs grade 1–5 were detected in both study groups. The mainly hematological AEs might be explained by incomplete extracorporeal filtration and delayed hepatic release of melphalan [9, 17]. Furthermore, leakages alongside the double balloon catheter (used to occlude the IVC) might increase systemic melphalan [12]. We detected a higher rate of leakages per patient in group^hemihep^ (3 leakages in 3/7 patients) than in group^noresection^ (3 leakages in 1/7 patients), indicating that postsurgical anatomy makes positioning of the balloons more challenging. Nonetheless, in all patients an appropriate occlusion of the IVC was achieved before administration of melphalan. The liver-related AEs (reflecting direct toxicity of melphalan to the hepatocytes) were insignificantly higher in group^hemihep^, presumably explained by the overall lower number of hepatocytes after hemihepatectomy. The monitored hematological and hepatic toxic effects were responsive to therapy and normalized towards the end of the observational period in both groups – indicating that reduced liver volume after hemihepatectomy does not impact long term results and underlining the safety of the procedure. Thus, the relevance of slightly more short-term toxicity-related AEs in group^hemihep^ seems negligible.

The clinically relevant AEs grade 3–4 recorded in our study were comparable to the results of recent studies. Kaydis et al. [21] examined the safety and efficacy of CS-PHP in 51 patients with metastasized UM receiving 134 CS-PHP. Comparable to our results, post-procedural hematological toxicities were common. Moreover, the AEs were comparable to those reported in the original Phase III study [9].

We found an equal percentage distribution of AEs grade 1–4 in group^hemihep^ and group^noresection^, but one death in group^hemihep^. The deceased patient underwent surgery 172 months after first diagnosis of UM. Relapse occurred with disseminated intrahepatic metastasis and a high tumor burden (LDH 1559 U/I, norm: ≤ 250 U/I). SD was the response after the first procedure. After the second CS-PHP, the patient developed a neutropenic bacterial peritonitis and subsequent septic shock with multi organ failure and deceased eight days after CS-PHP. Of note, one patient in group^noresection^ with high pre-interventional LDH (1064 U/I) suffered from pancytopenia and sepsis and deceased 2.8 months after the first and only CS-PHP. In both cases, the adverse outcome was most likely related to high tumor burden, which has been described to have a negative correlation to survival [11]. Both patients were aware of their high-risk profile and were treated due to a strong therapeutic wish of the patients. Consequently to these events, further patients with a high tumor burden were more carefully discussed in our institution.

There are several limitations to this study. We performed a single center study including only a small number of patients. Patient data was retrospectively evaluated for complications and adverse events. As a transregional center, we treat patients from across Germany and some patients received their follow up examinations outside our center. As a result, 19% of laboratory data were unavailable for day 21–45 of the observational period. Nevertheless, we acquired the laboratory results for up to 7 days for all treatments. According to other studies, this might be adequate to assess the toxicity of melphalan [11]. Overall, the number of interventions and the number of patients included in this study is limited. The patients included in group^noresection^ did not match the patients in group^hemihep^ in all characteristics due to a lack of possible options, as CS-PHP is a rarely conducted procedure for selected patients only. This might lead to a limited comparability.

The severity of adverse events following chemosaturation percutaneous hepatic perfusion in patients after right hemihepatectomy was comparable to a matched group without prior liver surgery. Therefore, chemosaturation percutaneous hepatic perfusion with melphalan might be safely performed in patients following hemihepatectomy.

## Abbreviations

AE: Adverse event
BAC: Best available care
CS-PHP: Chemosaturation percutaneous hepatic perfusion
CTCAE: Common terminology criteria for adverse events
ECOG: Eastern cooperative oncology group
iCCA: Intrahepatic cholangiocarcinoma
INR: International normalized ratio
IVC: Inferior vena cava
LDH: Lactate dehydrogenase
MELD: Model for end-stage liver disease
OS: Overall survival
RECIST: Response evaluation criteria in solid tumors
SD: Stable disease
TARE: Transarterial radioembolization
UM: Uveal melanoma
